# Use of human fibrin glue (Tisseel) versus suture during transvaginal natural orifice ovarian cystectomy of benign and non-endometriotic ovarian tumor: a retrospective comparative study

**DOI:** 10.1186/s12893-021-01061-1

**Published:** 2021-01-21

**Authors:** Yu-Ying Su, Yu-Shan Lin, Lan-Yan Yang, Yu-Bin Pan, Yi-Ting Huang, Cindy Hsuan Weng, Kai-Yun Wu, Chin-Jung Wang

**Affiliations:** 1 Department of Obstetrics and Gynecology, Chang Gung Memorial Hospital at Linkou, Kweishan, Taoyuan Taiwan; 2 Clinical Trial Center, Chang Gung Memorial Hospital Linkou Medical Center, Taoyuan, Taiwan; 3 Chang Gung University College of Medicine, Taoyuan, Taiwan

**Keywords:** Adnexal mass, Cystectomy, Laparoscopy, Transvaginal natural orifice surgery, Tisseel

## Abstract

**Background:**

To evaluate the use of a human fibrin glue (Tisseel) for minor bleeding control and approximation of ovarian defect during transvaginal natural orifice ovarian cystectomy (TNOOC) of benign and non-endometriotic ovarian tumors.

**Methods:**

A total of 125 women with benign and non-endometriotic ovarian tumors who underwent TNOOC between May 2011 and January 2020: 54 with the aid of Tisseel and 71 with traditional suture for hemostasis and approximation of ovarian defect. Surgical outcomes such as length of surgery, operative blood loss, postoperative pain score, and postoperative hospital stay were recorded. Before and immediately (10 days) and at 6 months after the procedure, serum anti-Müllerian hormone (AMH) levels were also determined.

**Results:**

Complete hemostasis and approximation of ovarian defect were achieved in all cases. No significant difference was noted in the operating time, operative blood loss, postoperative pain scores after 12, 24 and 48 h, length of postoperative stay, and baseline AMH levels between the two groups. The operation did not have a negative effect on the immediate and 6-month postoperative AMH levels in the suture group. However, the decline in the AMH levels was significant immediately after surgery in the Tisseel group, nevertheless, no significant difference was noted in the AMH levels at 6 months (3.3 *vs.* 1.7 mg/mL; *p* = 0.042, adjusted *p* = 0.210).

**Conclusion:**

The use of Tisseel in TNOOC of benign and non-endometriotic ovarian tumors without suturing the ovarian tissue is clinically safe and feasible.

## Background

An ovarian mass is a common gynecologic problem. The occurrence rate reported from a random sample of premenopausal women was 6.6% [1]. Most ovarian tumors (80–85%) are benign [2], and ovarian cystectomy is suggested to make a precise diagnosis, prevent potential torsion or rupture, and preserve the ovarian tissue for women in reproductive age [3, 4]. With the development of laparoscopic techniques in the past decades, today, the majority of benign ovarian tumors are managed in this manner [5, 6].

Benign ovarian tumors, even a large one (> 8 cm), are usually associated with simple intra-abdominal conditions (free from adhesions and maintaining the relative position among adjacent organs) compared with malignance, abscess, and endometriotic tumors. The four major steps of ovarian cystectomy are mass excision, hemostasis, approximation of ovarian defect, and extraction of surgical specimen. Since laparoscopic treatment is the mainstream nowadays for benign ovarian tumors, the transvaginal endoscopic surgery-assisted approach (TESA) can complete the aforementioned works simultaneously and provide excellent cosmetic effect (no scars on the abdomen) and fast recovery for woman with sexual experience [7–11].

Although conventional surgical instruments can be used in the TESA, the nature of vaginal surgery, limited operating space, may increase the difficulty of achieving good hemostasis. The fibrin sealant Tisseel (Baxter Healthcare Corporation, Deerfield, IL) is a two-component product that consists of a thrombin solution (human thrombin and calcium chloride) and a sealer protein solution (human fibrinogen and aprotinin). It is used for improving hemostasis, gluing or sealing tissues, and enhancing wound healing [12]. Our prior study demonstrated the safety and feasibility of using Tisseel in laparoscopic excision of ovarian endometrioma [13]. Therefore, in the present study, we describe a novel simple technique for Tisseel application during transvaginal natural orifice ovarian cystectomy (TNOOC) for benign and non-endometriotic ovarian tumors. Furthermore, a comparison of the surgical outcomes and ovarian reserve marker, anti-Müllerian hormone (AMH) levels, of these patients with other TNOOC patients who underwent traditional suture hemostasis and approximation is performed.

## Methods

**Fig. 1:**
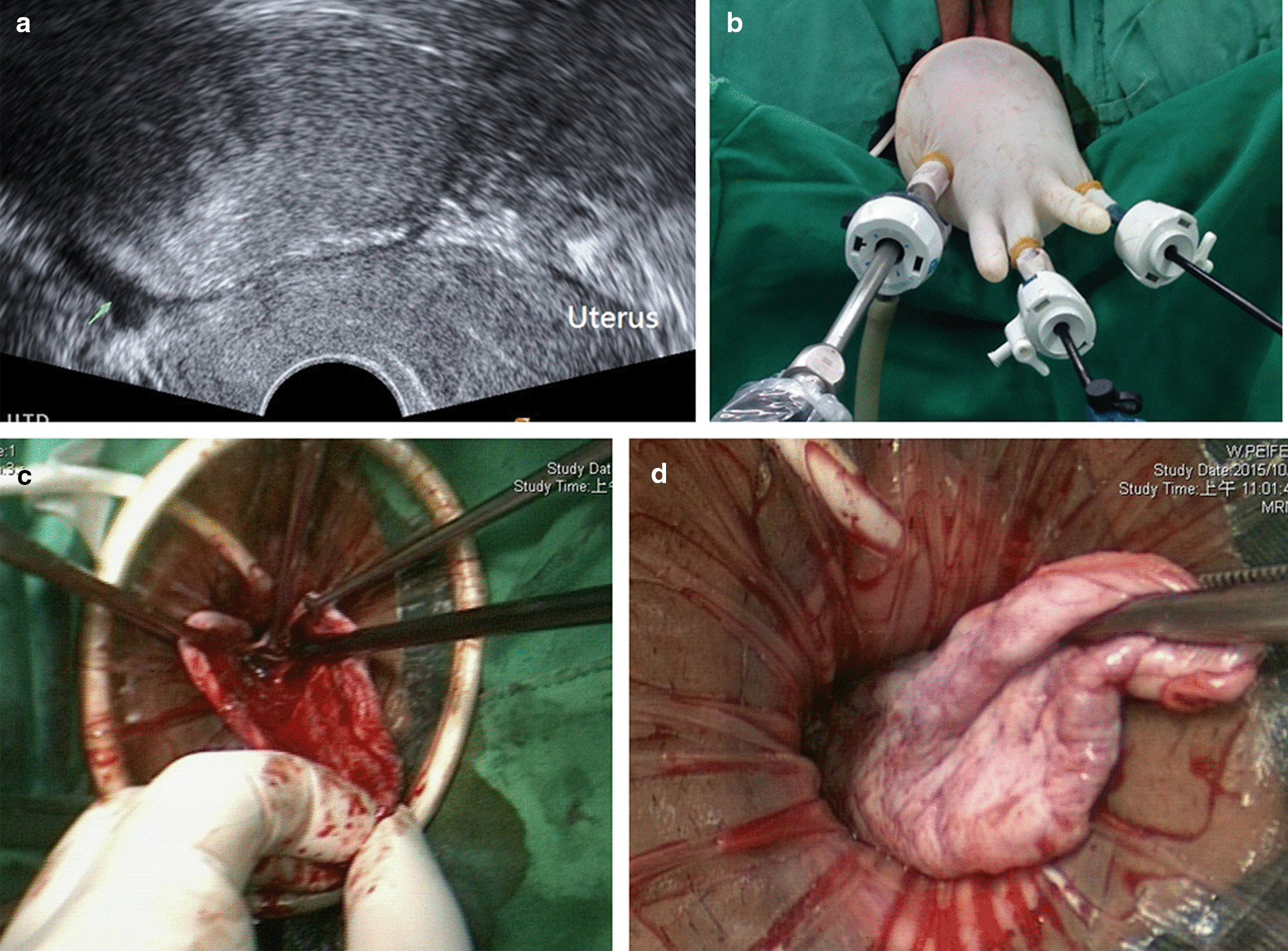
**a** Transvaginal ultrasonography showing a well-defined solid tumor (arrow) measuring 9.49 × 5.39 cm in the right adnexal area. **b** Intraoperative images showing the transvaginal natural orifice transluminal endoscopic portal. **c** Tisseel is applied on the inner ovarian surface defects with a dual lumen applicator. **d** The ovarian defect is approximated with an atraumatic forceps for 3 min

This was a single-institution retrospective study conducted at a tertiary-care university hospital. From May 2011 to January 2020, 485 patients with non-endometriotic ovarian tumors were scheduled to undergo laparoscopy, which was performed by one of the authors (CJW). A total of 54 sex-experienced women (median age, 32.5 years; range, 18–44) underwent TNOOC with the aid of Tisseel. Moreover, as a contemporary cohort comparison, data of 71 women who underwent the same type of surgery with traditional suture, without Tisseel, to achieve hemostasis and approximation of ovarian defect were also retrospectively studied; this surgery was also performed by the same operator (CJW). Before the operation, medical history taking, pelvic examination, transvaginal sonography (Fig. 1a), and/or computed tomography to evaluate tumor characteristics were performed in each patient. The primary outcomes were length of surgery, operative blood loss, and postoperative hospital stay. The secondary outcomes included postoperative pain score and baseline serum AMH levels and declined over immediate (10 days) postoperative and 6-month follow-up periods. The study protocol was reviewed and approved by the local Institutional Review Board.

### Operative procedures

The procedures for TNOOC have been previously described in detail [10]. In brief, operations were performed with general endotracheal anesthesia in the lithotomy and Trendelenburg positions. Both legs of the patient were protected by elastic bandages, and a Foley catheter was inserted for constant urinary drainage. Posterior colpotomy was then created using traditional vaginal surgical techniques. A working port system with a wound retractor (Alexis, Small; Applied Medical Resources Corp., Rancho Santa Margarita, CA, USA) attached with a surgical glove was then inserted transvaginally through the colpotomy incision. One 10-mm and two 5-mm sheaths were inserted through cut edges of the thumb and middle and little fingertips of the glove and tied with elastic bandage to prevent gas leakage. Once the working port system placement was completed, traditional laparoscopic instruments including a 0^0^ telescope were used, and the procedures were started (Fig. 1b). After identifying the location of the mass, a grasping forceps was used to pull the mass into the posterior Douglas pouch. The glove was removed at this time, but the wound retractor was left in place. A sharp cortical incision was made using a 15-blade scalpel and a cleavage plane was identified. Controlled drainage of the cystic component was then performed by inserting an 18-gauge needle connected to a suction apparatus. After decompression, the ovarian tumor was pulled into the vagina. Ovarian cystectomy was then performed transvaginally in a traditional manner by using conventional vaginal surgical instruments. No endoscopic electrosurgery device (such as bipolar cautery or monopolar scissors) was used. After tumor removal, hemostasis was checked in the ovarian defect and approximated using a 3–0 monofilament poliglecaprone 25 suture (Monocryl; Ethicon Inc., Somerville, NJ, USA). For the optimal use of Tisseel, the inner surfaces of healthy ovarian remnants were dried as much as possible using a gauze. Tisseel was applied with a dual lumen applicator (Fig. 1c) to achieve a uniform coating on the inner surface of the ovarian defect. Thereafter, the edges of the ovarian defect were approximated with the surgeon’s hand or atraumatic forceps for 3 min (Fig. 1d). After the aforementioned procedures, the ovarian remnants were then restored to their normal anatomic position, and the working port system and pneumoperitoneum were reestablished. All surgical sites were inspected to ensure complete hemostasis. Finally, the working port system was removed, and the colpotomy was closed with 2–0 polyglycolic acid sutures (Vicryl; Ethicon Inc., Somerville, NJ, USA).

### Statistical analysis

All continuous variables are expressed as medians and ranges. Continuous variables were compared using the Mann–Whitney *U* test, while categorical values were compared using Pearson’s chi-square or Fisher’s exact test. Moreover, the Wilcoxon signed rank test was used to compare the pre- and postoperative AMH values. A Bonferroni adjustment was used to adjust for multiple group comparisons. All calculations were performed using the SPSS 21.0 statistical package (IBM, Armonk, NY, USA). A probability value of < 0.05 was considered statistically significant.

## Results

All procedures were completed transvaginally, and no laparoscopy conversions in the study were necessary. In the study population (n = 54), 30 non-endometriotic ovarian tumors were located on the left side, 22 on the right side, and 2 on both sides. The median diameter of the main ovarian mass was 8 cm, with a range of 5–17 cm. The baseline characteristics of patients are summarized in Table 1. The median number of vaginal delivery was 0 (range, 0–3). The median operating time and operative blood loss were 43.5 min (range, 29–180 min) and 20 mL (range, 2–100 mL), respectively. Postoperative hospital stay was 1 day (range, 1–2 days) (Table 2). No major complications including bladder, bowel, or ureter injury occurred.

**Table 1:** Baseline characteristics of enrolled patients who underwent transvaginal natural orifice cystectomy with (group A) and without Tisseel (group B)

	Group A (n = 54)	Group B (n = 71)	*P*
Patients			
Age (years)	32.5 (18–44)	34.0 (20–48)	0.089
Body mass index (kg/m^2^)	20.9 (15.7–36.8)	21.2 (16.3–40.3)	0.411
Vaginal delivery	0 (0–3)	0 (0–4)	0.032
Yes	16 (29.6)	33 (46.5)	0.056
No	38 (70.4)	38 (53.5)	
Bilaterality of masses^a^			0.698
Unilateral	52 (96.3)	66 (93.0)	
Right side	22 (40.7)	36 (50.7)	0.269
Left side	30 (55.6)	30 (42.3)	0.140
Bilateral sides	2 (3.7)	5 (7.0)	
Main mass diameter (cm)	8 (5–17)	7 (5–19)	0.292
CA125 before operation (U/mL)	17.7 (5–56)	15.7 (6–53)	0.134

Values are given as median (range) or count (%). P values were measured using the Mann–Whitney U test and Pearson’s chi-square or Fisher’s exact test, as appropriate

*CA125*  cancer antigen

^a^Determined with preoperative transvaginal pelvic ultrasonography

**Table 2:** The comparisons of outcome variables between patient cohorts who underwent transvaginal natural orifice cystectomy with (group A) and without Tisseel (group B)

	Group A (n = 54)	Group B (n = 71)	*P*-value	Adjust *P*-value
Operating time (m)	43.5 (29–180)	40.0 (25 – 105)	0.029	0.203
Blood loss (mL)	20.0 (2–100)	20.0 (5—300)	0.282	0.964
Postoperative pain score				
12 h postoperatively^a^	2.0 (0–9)	3.5 (0–10)	0.049	0.294
24 h postoperatively^b^	1.0 (0–5)	2.5 (0–8)	0.095	0.475
36 h postoperatively^c^	0 (0–3)	0 (0–3)	0.647	0.964
Postoperative stay (days)	1 (1–2)	1 (1–3)	0.385	0.964
Pathological diagnoses			0.241	0.964
Mature teratoma^d^	35 (64.8)	47 (66.2)	0.872	
Fibroma	1 (1.9)	0 (0)	0.432	
Follicular cyst	0 (0)	3 (4.2)	0.258	
Simple cyst	2 (3.7)	3 (4.2)	1.000	
Mucinous cystadenoma	8 (14.8)	14 (19.7)	0.476	
Serous cystadenoma^e^	8 (14.8)	4 (5.6)	0.084	

Values are given as median (range) or count (%)

*P*-values adjusted by the Holm–Bonferroni method

^a^46 patients in group A and 22 patients in group B have postoperative 12-h data

^b^35 patients in group A and 17 patients in group B have postoperative 24-h data

^c^17 patients in group A and 12 patients in group B have postoperative 36-h data

^d^Mature teratoma includes dermoid cyst and struma ovarii

^e^Serous cystadenoma includes serous cystadenofibroma

A cohort of patients who underwent TNOOC using suture during the same period was identified (n = 71). When the pure suture cohort was compared with the group who had TNOOC with Tisseel, no significant difference was noted with respect to age, body mass index, bilaterality of ovarian tumors, main tumor size, and preoperative levels of serum cancer antigen 125. The pure suture group had more number of vaginal delivery than the Tisseel group (*p* = 0.032), but no significant difference in the history of vaginal delivery was noted between the two groups (*p* = 0.056) (Table 1).

The detailed data of operative outcomes are listed in Table 2. Although a longer operating time (43.5 *vs.* 40.0 min; *p* = 0.029) and reductions in postoperative pain after 12 h (2.0 *vs.* 3.5; *p* = 0.049) were observed in the Tisseel group. However, they did not show statistical significance after the Bonferroni adjustment for multiple comparisons. No significant difference was noted in blood loss, postoperative stay, and the postoperative pain scores after 24 and 48 h between the two groups. Benign teratoma including dermoid cyst and strumsa ovarii was the major pathology in both groups (Table 2).

The ovarian reserve marker, AMH levels, was analyzed and showed no significant differences between the two groups at baseline and immediate postoperative phase (10 days after operation). Women with Tisseel had significantly higher AMH levels than those in the pure suture group at 6 months (3.3 *vs.* 1.7 mg/mL; *p* = 0.042) but lost its significance after adjustment (adjusted *p* = 0.210). The operation did not have a negative effect on the immediate (10 days) and 6-month postoperative AMH levels in the pure suture group. However, using Tisseel for hemostasis and approximation, there was a significant decline in ovarian reverse at 10 days after operation (*p* = 0.002, adjusted p = 0.004). The levels rose by the sixth month without significance than at baseline (*p* = 0.719) (Table 3). The median decline in ovarian reserve in women who underwent TNOOC with Tisseel was − 0.29 ng/mL (range, − 2.96–1.37) at 10 days and − 0.14 ng/mL (range, − 5.49–6.16) at 6 months. This was correlated with a decrease of 10.4% and 5.0%, respectively. In the pure suture group, the median decline in ovarian reserve was − 0.36 ng/mL (range, − 2.3–0.79) at 10 days and − 0.44 ng/mL (range, − 3.28–1.06) at 6 months. This was correlated with a decrease of 14.4% and 17.6%, respectively.

**Table 3:** The comparisons of the anti-Müllerian hormone (AMH) levels between patient cohorts who underwent transvaginal natural orifice cystectomy with (group A) and without Tisseel (group B)

Visit	Statistics	Group A (mg/mL)	Group B (mg/mL)	*P*-value	Adjust *P*-value
Baseline	N Median (range)	51 2.8 (0.26–12.7)	20 2.5 (0.15–12.3)	0.292^*^	*0.584*
Immediate (10 days postoperatively)	N Median (range)	41 3.0 (0.3–13.3)	18 2.1 (0–8.1)	0.056^*^	0.224
Change from baseline to 10 days	N *P-value* Adjust *P*-value	41 0.002^†^ 0.004	15 0.156^†^ 0.156	0.948^*^	0.948
6 months	N Median (range)	27 3.3 (0.07–18.2)	14 1.7 (0–6.8)	0.042^*^	0.210
Change from baseline to 6 months	N *P-value* Adjust *P*-value	27 0.719^†^ 0.719	12 0.06^†^ 0.120	0.188^*^	0.564

*P*-values adjusted by the Holm–Bonferroni method

^*^Mann–Whitney U test

^†^Wilcoxon signed rank test

## Discussion

Benign ovarian tumors, particularly large ones, usually have a phenomenon of deep embedding in the cul-de-sac [9]. Moving tumors to the top position of the pelvic cavity and then continuing with the remaining procedures is the first step of ovarian surgery [3, 6, 10, 14]. However, avoiding spillage of tumor content is sometimes difficult for a large tumor at this phase during laparoscopy. On the contrary, almost all tumors were visible at once after posterior colpotomy [9]. The remaining tumor floating in the pelvis can be pulled into the cul-de-sac through the TNOOC surgical platform. Controlled drainage and decompression of the tumor were then performed to permit cystectomy using conventional vaginal surgical instruments with ease. Meanwhile, the surgical specimens were also extracted. The major advantages of TNOOC include excision of tumor and removal of specimens completed simultaneously and the hidden incision scar in the vagina [10].

The feature that caused the greatest difficulty of TNOOC was stanching bleeding from the ovarian hilum. The bleeding points in the high site of the pelvis, relatively limited operative field, and narrow field of vision made the hemostasis more challenging for surgeons. The feasibility of transvaginal surgery is influenced by the width of the operative field, that is, depending on the number of vaginal delivery of the woman. From this technical point of view, it can be explained why women who underwent TNOOC with suture had more number of vaginal delivery than those with Tisseel (*p* = 0.032). It is noteworthy, however, that nulliparity was observed in the majority of both groups (70.4% and 53.5% in the Tisseel and suture groups, respectively), but no significant difference in the history of vaginal delivery was noted between the two groups (*p* = 0.056). The results demonstrated nulliparity did not represent an absolute contraindication where there was an adequate vaginal caliber.

Theoretically speaking, the operating time of TNOOC with Tisseel will be shorter than with suture. However, our data did not show the significance after the adjustment. This result revealed 2 points. First, as mentioned earlier, the parity of vaginal delivery demonstrated its facilitating influence in vaginal surgery. Second, the proper application of Tisseel (drying the site of application as much as possible and allowing at least 2 min after application to achieve sufficient polymerization) was not as fast as imagined. A proficient gynecologist can efficiently and easily perform either TNOOC with Tisseel or with traditional instruments and techniques despite of women with or without history of vaginal delivery.

The Tisseel group had lower postoperative abdominal pain at 12 h; however, no significant difference was achieved, which may result from using the fibrin sealant instead of suture for hemostasis to decrease ischemic reaction in the ovarian hilum and less manipulation during the whole procedures. However, the pain scores were similar at 36 h after surgery. Possible explanations for this point are that the blood supply of the healthy ovarian remnants resumed gradually, and inactivation and desensitization of nociceptors occur at this time period [15, 16]. The similar finding is also revealed by a clinical study on transvaginal endoscopic salpingectomy for ectopic pregnancy reported by Xu et al. [17].

No previous study has directly compared ovarian reserve after hemostasis by suture and fibrin sealant during ovarian cystectomy for managing benign ovarian tumors. Several studies reported a significant decrease in the AMH levels was observed in the endometriotic and non-endometriotic tumors after the procedure with suture for hemostatic control at the short-term (1–3 months) follow-up. The values would gradually recover but remained depressed from baseline and were no longer significantly different at 6 months after surgery [18–20]. Our results conformed to these findings. However, it is interesting that a significant decline in the AMH levels was revealed immediately (10 days) after surgery in patients with Tisseel but was not observed in the suture group. This could be explained partly by the fact that there was no thermal damage in both groups, as no endoscopic electrosurgery device was used in either groups. And partly by the pharmacodynamic properties of Tisseel: the fibrin clots containing collagen-rich granulation tissue are markedly absorbed after 14 days [12]. Excess fibrin clot thickness in the Tisseel group may negatively interfere with tissue healing, leading to subsequent decline in the AMH levels.

The selected cases in this study were suspected benign and non-endometriotic ovarian tumors. However, potentially encountering an unexpected ovarian malignancy during surgery is still a major issue. When laparoscopic surgery is used for adnexal masses with complex aspects on sonography, the probability of falling on a malignancy may be as high as 11 to 19%. On the contrary, limiting laparoscopy to patients with simple cysts without solid component, reduces the probability of meeting an unexpected malignancy to 0–2.5% [21]. The International Ovarian Tumor Analysis (IOTA) group ultrasound rules for ovarian masses are a simple set of ultrasound findings that can improve the diagnosis of ovarian cancer. By simply using the IOTA rules, an accurate result can be obtained in most cases with a sensitivity of 92% and a specificity of 96% [22]. Thus, initial evaluation of ovarian masses by sonography is crucial. Clinicians can minimize the risk of encountering an unexpected finding during surgery by performing adequate preoperatory evaluation.

Avoiding tumor spillage during surgery, especially in unexpected ovarian malignancies, is another concern. Unintended rupture of tumor sac may spread its contents and possibly cause chemical peritonitis, subsequent adhesion formation, upstaging of the ovarian cancer from stage IA to stage IC, or the port-site metastasis, and thus, negatively impact disease-free survival [21]. With the techniques demonstrated in the study, intraperitoneal spillage was avoided by controlled drainage. In addition, the application of a wound retractor extended the colpotomy incision and reduced the risk of contamination by the contents of the mass [10].

A strong point of the present study was that all procedures were performed by a single surgeon (CJW), making the quality of surgery consistent. The comparisons between the two groups can reflect the exact differences by minimizing the influence of surgical skills. Using data from operations performed by a single surgeon was also one of the limitations of this study. That is, the results may not be applicable to the entire gynecologic public. Further, we did assess the surgical impacts on the postoperative pain scores and serum AMH levels. However, the nature of this retrospective study and the small group of patients who completed all follow-ups made it difficult to demonstrate the accurate conditions.

## Conclusions

In short, our study describes a novel straightforward technique for the management of benign and non-endometriotic ovarian tumors. The proposed approach is combining the use of a minimally invasive endoscopic technique with vaginal surgery without requiring special surgical instrumentation. The application of a fibrin sealant may facilitate in dealing with blood oozing from the ovarian hilum and approximating the ovarian defect. Generally, the process of approximating the ovary via suture is dependent on and influenced by a surgeon’s technical finesse; whereas the same process delivered via Tisseel using an applicator may be more user-friendly and may cater to a wider group of users to achieve uniform results and the end goal of restoring ovarian anatomy. In addition, hiding the incision in the vagina more meets the merit of minimally invasive surgery. Surgeons still need to well select patients (clinically presumed benign tumor, no intra-abdominal and pelvic adhesions, and adequately sized vagina) to undergo TNOOC with Tisseel. However, this product is not covered by public health insurance in Taiwan, and surgeons need to be aware of the additional cost. At this time, this technique shows its potential for wider implementation in the field of laparoscopic surgery. Further randomized prospective trials are needed to achieve a solid conclusion.

## Data Availability

The datasets used and analyzed during the current study are available from the corresponding author on reasonable request.

## Abbreviations

AMH: Anti-Müllerian hormone
CA125: Cancer antigen 125
TESA: Transvaginal endoscopic surgery-assisted approach
TNOOC: Transvaginal natural orifice ovarian cystectomy
